# Cocirculation of Genetically Distinct Highly Pathogenic Avian Influenza H5N5 and H5N1 Viruses in Crows, Hokkaido, Japan

**DOI:** 10.3201/eid3009.240356

**Published:** 2024-09

**Authors:** Yik Lim Hew, Takahiro Hiono, Isabella Monne, Kei Nabeshima, Saki Sakuma, Asuka Kumagai, Shunya Okamura, Kosuke Soda, Hiroshi Ito, Mana Esaki, Kosuke Okuya, Makoto Ozawa, Toshiyo Yabuta, Hiroki Takakuwa, Linh Bao Nguyen, Norikazu Isoda, Kohtaro Miyazawa, Manabu Onuma, Yoshihiro Sakoda

**Affiliations:** Hokkaido University, Sapporo, Japan (Y.L. Hew, T. Hiono, L.B. Nguyen, N. Isoda, Y. Sakoda);; Istituto Zooprofilattico Sperimentale delle Venezie, Padova, Italy (I. Monne);; National Institute for Environmental Studies, Tsukuba, Japan (K. Nabeshima, M. Onuma);; National Agriculture and Food Research Organization, Tsukuba (S. Sakuma, A. Kumagai, K. Miyazawa);; Tottori University, Tottori, Japan (S. Okamura, K. Soda, H. Ito);; Kagoshima University, Kagoshima, Japan (M. Esaki, K. Okuya, M. Ozawa);; Kyoto Sangyo University, Kyoto, Japan (T. Yabuta, H. Takakuwa)

**Keywords:** highly pathogenic avian influenza virus, H5N5, subtype, H5N1, influenza, respiratory infections, viruses, zoonoses, crows, Japan

## Abstract

We isolated highly pathogenic avian influenza (HPAI) H5N5 and H5N1 viruses from crows in Hokkaido, Japan, during winter 2023–24. They shared genetic similarity with HPAI H5N5 viruses from northern Europe but differed from those in Asia. Continuous monitoring and rapid information sharing between countries are needed to prevent HPAI virus transmission.

H5 highly pathogenic avian influenza viruses (HPAIVs) of the A/goose/Guangdong/1/1996 lineage have diversified into multiple clades, threatening wild birds and poultry worldwide. Clade 2.3.4.4b HPAIVs have been consistently isolated in Asia and Europe since 2016 (*1**–**3*) and expanded further to North America in late 2021 (*4*). The global circulation of H5 HPAIVs over a relatively short time highlights the pivotal role of migratory birds in virus dissemination (*5*). H5 HPAIVs in clade 2.3.4.4 frequently acquire the neuraminidase (NA) gene from locally circulating low pathogenicity avian influenza viruses (LPAIVs), which often infect waterfowl, leading to the generation of novel H5Nx reassortant viruses, such as H5N2, H5N6, and H5N8 (*6*).

During the winter seasons 2021–22 and 2022–23, Hokkaido, located in the northernmost part of Japan, experienced HPAIV outbreaks driven by bird migration that substantially affected poultry and other resident birds. Those viruses clustered in the group 2 (G2) d subgroup within clade 2.3.4.4.b, which has multiple subgroups, G2a–e, and shared a common ancestor with HPAIVs detected in Europe in late 2020 (*7*). HPAIV subgroup G2d might have undergone intercontinental transmission from Europe to Japan (*8*,*9*). During winter 2023–24, H5N5 HPAIVs were detected in a crow flock in Hokkaido, and further monitoring revealed cocirculation of 2 distinct viruses in the crow population. We investigated the genetic origin and antigenicity of H5N5 HPAIVs isolated in Hokkaido.

## The Study

We conducted passive surveillance of HPAIV infections in wild birds in a public garden in Sapporo, the prefectural capital of Hokkaido, Japan; ≈2,000 crows flock together during winter and are observed by garden staff. We isolated viruses from tracheal and cloacal swab samples collected from dead crows in the garden by inoculating 10-day-old embryonated eggs; we confirmed results by using reverse transcription PCR (Appendix). On November 23 and 24, 2023, we isolated H5N1 HPAIVs from 2 dead large-billed crows (*Corvus macrorhynchos*), designated as A/large-billed crow/Hokkaido/B067/2023 (H5N1) and A/large-billed crow/Hokkaido/B068/2023 (H5N1). The hemagglutinin (HA) gene sequences from those 2 H5N1 HPAIVs indicated they clustered with the G2d subgroup of HPAIVs found in Hokkaido during the winter seasons 2021–22 and 2022–23. In contrast, HA genes of 3 H5 HPAIVs isolated from dead crows on January 8–11, 2024, were closely related to the G2a subgroup of H5N5 HPAIVs found in northern Europe and North America. Subsequent whole-genome sequencing analysis of the 3 G2a HPAIVs confirmed their subtype was H5N5; we named them A/large-billed crow/Hokkaido/B073/2024 (H5N5), A/large-billed crow/Hokkaido/B074/2024 (H5N5), and A/crow/Hokkaido/B075/2024 (H5N5) (Table 1).

**Table 1:** H5 viruses isolated in Hokkaido and Kumamoto, Japan, in winter 2023–24 in study of cocirculation of genetically distinct highly pathogenic avian influenza H5N5 and H5N1 viruses in crows*

Virus name	Subgroup	Date†	City/town	Latitude	Longitude	Accession no.
A/large-billed crow/Hokkaido/B067/2023 (H5N1)	G2d	2023 Nov 23	Sapporo	43°03′50”N	141°20′35”E	EPI_ISL_18591747
A/large-billed crow/Hokkaido/B068/2023 (H5N1)	G2d	2023 Nov 24	Sapporo	43°03′50”N	141°20′35”E	EPI_ISL_18594618
A/large-billed crow/Hokkaido/0112F066T/2023 (H5N5)	G2a	2023 Dec 19	Erimo	42°00′59”N	143°08′53”E	EPI_ISL_18837770
A/large-billed crow/Hokkaido/0112F066C/2023 (H5N5)	G2a	2023 Dec 19	Erimo	42°00′59”N	143°08′53”E	EPI_ISL_18838019
A/large-billed crow/Hokkaido/B073/2024 (H5N5)	G2a	2024 Jan 8	Sapporo	43°03′50”N	141°20′35”E	EPI_ISL_18792212
A/large-billed crow/Hokkaido/B074/2024 (H5N5)	G2a	2024 Jan 9	Sapporo	43°03′50”N	141°20′35”E	EPI_ISL_18830859
A/crow/Hokkaido/B075/2024 (H5N5)	G2a	2024 Jan 11	Sapporo	43°03′50”N	141°20′35”E	EPI_ISL_18830860
A/large-billed crow/Hokkaido/B076/2024 (H5N1)	G2d	2024 Jan 12	Sapporo	43°03′50”N	141°20′35”E	EPI_ISL_18830861
A/large-billed crow/Hokkaido/B078/2024 (H5N1)	G2d	2024 Jan 17	Sapporo	43°03′50”N	141°20′35”E	EPI_ISL_18876661
A/carrion crow/Hokkaido/B079/2024 (H5N1)	G2d	2024 Jan 18	Sapporo	43°03′50”N	141°20′35”E	EPI_ISL_18876662
A/large-billed crow/Hokkaido/B080/2024 (H5N1)	G2d	2024 Jan 22	Sapporo	43°03′50”N	141°20′35”E	EPI_ISL_18932042
A/carrion crow/Hokkaido/B081/2024 (H5N1)	G2d	2024 Jan 26	Sapporo	43°03′50”N	141°20′35”E	EPI_ISL_18876663
A/large-billed crow/Hokkaido/B104/2024 (H5N5)	G2a	2024 Feb 20	Sapporo	43°03′50”N	141°20′35”E	EPI_ISL_19033207
A/large-billed crow/Hokkaido/B120/2024 (H5N5)	G2a	2024 Mar 16	Sapporo	43°03′50”N	141°20′35”E	EPI_ISL_19055087
A/large-billed crow/Hokkaido/B157/2024 (H5N5)	G2a	2024 Apr 30	Sapporo	43°03′50”N	141°20′35”E	EPI_ISL_19174744
A/peregrine falcon/Kumamoto/4301C001/2024 (H5N5)	G2d	2024 Jan 16	Kumamoto	32°56′07”N	130°33′45”E	EPI_ISL_18876660

*Accession numbers are from GISAID (https://www.gisaid.org). †Date of sample collection.

We phylogenetically analyzed virus isolates along with reference sequences obtained from GISAID (https://www.gisaid.org); the HA genes of H5N5 HPAIVs isolated in Hokkaido diverged considerably from HPAIVs isolated in Japan during winter 2020–21 (*10*), forming a distinct branch within the G2a subgroup (Figure). In addition, the other gene segments of H5N5 HPAIVs from Hokkaido were genetically distant from those in HPAIV strains isolated in Japan during winter 2021–22 (Appendix Figures 1–6). BLAST (https://blast.ncbi.nlm.nih.gov) analysis of sequences from GISAID revealed that all 8 gene segments of H5N5 HPAIVs from Hokkaido were very close (genetic similarity >99%) to H5N5 HPAIVs detected in northern Europe since 2022, in contrast to those from North America (Table 2), suggesting a low possibility of virus transmission from North America. H5N5 HPAIVs from Hokkaido shared a common ancestor with H5N5 HPAIV from Europe assigned the genotype EA-2021-I by the European Food Safety Authority (*11*). Parent strains of H5N5 HPAIVs from Europe, represented by A/swan/Rostov/2299-2/2020 (H5N5), were proposed to originate in western Russia during autumn 2020. Those viruses underwent genetic evolution via reassortment events involving H5N8 HPAIVs circulating in Europe since 2018 (*12*) and the N5 NA gene derived from concurrently circulating LPAIVs (*13*). H5N5 HPAIVs reported in northern Europe during 2022–2023 exhibited specific genetic differences compared with H5N5 HPAIVs detected in Europe during autumn 2020, particularly in the N5 NA gene. Those differences included a 66-bp nucleotide deletion within the N5 NA gene, which we also observed in the H5N5 HPAIVs from Hokkaido. Truncation of the NA stalk has been attributed to the adaptation of those viruses from wild birds to *Galliformes* spp. birds (*14*). However, most H5N5 HPAIV infections in Europe were detected in wild birds, and no cases have been detected in *Galliformes* spp. birds since 2022 (*15*). Further investigation is needed to clarify whether NA stalk truncation affects pathogenesis of H5N5 HPAIVs.

**Figure:**
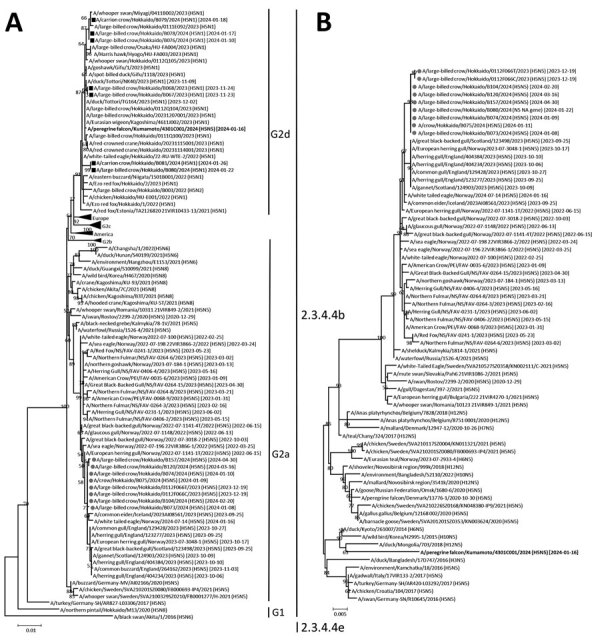
Phylogenetic analysis of genetically distinct highly pathogenic avian influenza H5N5 and H5N1 viruses isolated in Japan in winter 2023–24. H5 hemagglutinin (A) and N5 neuraminidase (B) gene segments of H5N5 highly pathogenic avian influenza viruses (HPAIVs) isolated in winter 2023–24 were compared with reference strains within clade 2.3.4.4b obtained from GISAID (https://www.gisaid.org). Squares indicate H5N1 and circles indicate H5N5 HPAIVs isolated from crows in Hokkaido in winter 2023–24. Bold text indicates the H5N5 HPAIV isolated from a peregrine falcon in Kumamato in the southern part of Japan in winter 2023–24. Trees were constructed by using the maximum-likelihood method and MEGA 7 software (https://www.megasoftware.net). Bootstrap values (>50%) from 1,000 replicates are indicated on nodes. Isolated viruses belonging to subgroups G1, G2a, and G2d and clade 2.3.4.4b are indicated. Dates after strain names indicate sample collection dates for HPAIV-infected animals. Scale bar indicates number of nucleotide substitutions per site.

**Table 2:** BLAST search results of H5N5 HPAIVs isolated in Japan in winter 2023–24 in study of cocirculation of genetically distinct H5N5 and H5N1 HPAIVs in crows, Hokkaido, Japan*

Virus name	Gene	Most homologous strain	Homology, %	Accession no.
A/large-billed crow/Hokkaido/B073/2024 (H5N5)†	PB2	A/Common eider/Iceland/2023AI08561/2023 (H5N5)	99.7	EPI2791002
	PB1	A/Common eider/ Iceland/2023AI08561/2023 (H5N5)	99.8	EPI2791003
	PA	A/Common eider/ Iceland/2023AI08561/2023 (H5N5)	99.7	EPI2791001
	HA	A/Common gull/England/129428/2023 (H5N5)	99.8	EPI2815885
	NP	A/Herring gull/England/404384/2023 (H5N5)	99.9	EPI2815894
	NA	A/Herring gull/England/404384/2023 (H5N5)	99.5	EPI2815900
	M	A/Herring gull/England/404384/2023 (H5N5)	99.9	EPI2815896
	NS	A/Herring gull/England/404384/2023 (H5N5)	99.9	EPI2815895
A/peregrine falcon/Kumamoto/4301C001/2024 (H5N5)‡	PB2	A/large-billed crow/Hokkaido/20231207001/2023 (H5N1)	99.9	EPI2898966
	PB1	A/large-billed crow/Hokkaido/20231207001/2023 (H5N1)	99.9	EPI2898967
	PA	A/large-billed crow/Hokkaido/0111Q100/2023 (H5N1)	99.8	EPI2841124
	HA	A/large-billed crow/Hokkaido/20231207001/2023 (H5N1)	99.9	EPI2898969
	NP	A/large-billed crow/Hokkaido/0112Q104/2023 (H5N1)	99.7	EPI2815894
	NA	A/Duck/Hokkaido/W24/ 2020 (H6N5)	99.1	EPI1896526
	M	A/large-billed crow/Hokkaido/20231207001/2023 (H5N1)	100	EPI2815896
	NS	A/large-billed crow/Hokkaido/20231207001/2023 (H5N1)	99.9	EPI2898973

*Gene segment sequences of 2 H5N5 HPAIVs were compared with those of other H5 virus sequences by using BLAST (https://blast.ncbi.nlm.nih.gov). Accession numbers are from GISAID (https://www.gisaid.org). HA, hemagglutinin; HPAIV, highly pathogenic avian influenza virus; M, matrix protein; NA, neuraminidase; NP, nucleoprotein; NS, nonstructural protein; PA, polymerase acidic; PB1, polymerase basic 1; PB2, polymerase basic 2. †H5N5 HPAIV isolated in Hokkaido in winter 2023–24. ‡H5N5 HPAIV isolated in Kumamoto Prefecture in winter 2023–24.

During winter 2023–24, we confirmed H5N5 HPAIV infections in wild birds, especially in crows, in Erimo (December 19, 2023, in south-central Hokkaido) and in Kushiro (January 18, 2024, in eastern Hokkaido); we also confirmed infection in a peregrine falcon (*Falco peregrinus*) in Tamana, Kumamoto Prefecture, Kyushu Island, on January 16, 2024. We classified the isolate from Tamana, A/peregrine falcon/Kumamoto/4301C001/2024 (H5N5), into the G2d subgroup according to its HA gene sequence, whereas its NA gene sequence was similar to that of LPAIVs isolated in East Asia (Table 2). Although this combination had not been observed in Japan, reassortment events between the HPAIV H5N1 G2d subgroup and LPAIVs have been documented (*9*). We detected H5N5 HPAIVs in Hokkaido in January 2024; a total of 85 crows were found dead in the Sapporo garden, 80 of which we diagnosed as HPAIV positive by the end of April. No HPAIVs were detected in birds within the garden after April 2024. The continuous detection of H5N5 HPAIVs in the Sapporo garden during January–April without unusual deaths of birds other than crows and multiple isolations of H5N5 HPAIVs in other areas of Hokkaido suggest the potential for widespread dissemination of H5N5 HPAIVs within the Hokkaido region. 

H5N1 G2d HPAIVs persisted in crows residing in the Sapporo garden even after the introduction of H5N5 G2a viruses, indicating concurrent circulation of genetically distinct viruses within a single crow population. Indeed, the average nanopore sequencing coverage for A/large-billed crow/Hokkaido/B080/2024 (H5N1) was 5497.4 reads for the N1 NA gene (G2d subgroup) and 1943.7 reads for the N5 NA gene (G2a subgroup) (Appendix Table 1). This observation suggests single hosts were co-infected with 2 viruses and reassortment occurred between viruses originating from geographically distant areas. Antigenic characterization of H5N5 HPAIVs suggested that the antigenicity of A/large-billed crow/Hokkaido/B073/2024 (H5N5) was close (2–4-fold differences in hemagglutination inhibition titers) to that of other H5 HPAIVs in the G2d subgroup (Table 3) despite their genetic diversity (Appendix Table 2).

**Table 3:** Cross-hemagglutination inhibition assay titers of H5 HPAIVs in study of cocirculation of genetically distinct H5N5 and H5N1 HPAIVs in crows, Hokkaido, Japan*

Tested virus	Subtype	Clade	Subgroup	Titers using antiserum against indicated H5 viruses							
				Cr/Hok/ B003/22	Ck/Hok/ E001/22	Ew/Hok/Q71/22	WTE/Hok/R22/22	Dk/VN/ HU16-DD3/23	Mdk/ DRC/ KAF1/17	Ck/Kum/1-7/14	Bs/Aki/1/16
Cr/Hok/ B073/24†	H5N5	2.3.4.4b	G2a	640	160	40	640	320	640	320	320
Cr/Hok/ B003/22	H5N2	2.3.4.4b	G2d	**640**	640	80	320	640	1,280	640	160
Ck/Hok/ E001/22	H5N1	2.3.4.4b	G2d	320	**640**	160	320	640	1,280	640	80
Ew/Hok/Q71/22	H5N1	2.3.4.4b	G2b	320	640	**320**	160	640	1,280	320	160
WTE/Hok/R22/22	H5N1	2.3.4.4b	G2d	640	640	160	**320**	640	2,560	640	80
Dk/VN/ HU16-DD3/23	H5N1	2.3.4.4b	G2c	640	320	80	640	**640**	320	320	80
Ck/Hok/ B102/23	H5N1	2.3.4.4b	G2c	640	320	80	320	1,280	160	160	40
Mdk/ DRC/ KAF1/17	H5N8	2.3.4.4b	NA	640	640	80	160	640	**1,280**	640	80
Fox/Hok/1/22	H5N1	2.3.4.4b	G2d	640	320	160	640	640	640	320	160
Cr/Hok/ B067/23	H5N1	2.3.4.4b	G2d	160	160	80	320	640	320	80	40
Np/Hok/M13/20	H5N8	2.3.4.4b	G1	640	640	20	160	640	2,560	640	40
Ck/Kum/1–7/14	H5N8	2.3.4.4c	NA	320	640	40	20	160	640	**640**	80
Bs/Akita/1/16	H5N6	2.3.4.4e	NA	160	320	20	80	160	320	160	**640**

*Antiserum against H5 virus isolates was used to test antigenicity of different H5Nx HPAIVs. Bold values indicate homologous titers. Each abbreviated name is defined as follows: Cr/Hok/B073/24 (H5N5), A/large-billed crow/Hokkaido/B073/2024 (H5N5); Cr/Hok/B003/22, A/large-billed crow/Hokkaido/B003/2022 (H5N2); Ck/Hok/E001/22, A/chicken/Hokkaido/HU-E001/2022 (H5N1); Ew/Hok/Q71/22, A/Eurasian wigeon/Hokkaido/Q71/2022 (H5N1); WTE/Hok/R22/22, A/white-tailed eagle/Hokkaido/22-RU-WTE-2/2022 (H5N1); Dk/VN/HU-16-DD3/23, A/duck/Vietnam/HU16-DD3/2023 (H5N1); Ck/Hok/B102/23, A/chicken/Hokkaido/HU-B102/2023 (H5N1); Mdk/DRC/KAF1/17, A/Muscovy duck/DR Congo/KAF1/2017 (H5N8); Fox/Hok/1/22, A/Ezo red fox/Hokkaido/1/2022 (H5N1); Cr/Hok/B067/23, A/large-billed crow/Hokkaido/B067/2023 (H5N1); Np/Hok/M13/20, A/northern pintail/Hokkaido/M13/2020 (H5N1); Ck/Kum/1–7/14, A/chicken/Kumamoto/1–7/2014 (H5N8); Bs/Akita/1/16, A/black swan/Akita/1/2016 (H5N6). HPAIV, highly pathogenic avian influenza virus; NA, not applicable. †H5N5 virus isolated in Hokkaido in winter 2023–24.

## Conclusions

We found that H5N5 HPAIVs consisting of unique gene constellations were likely introduced into Japan through a step-by-step bird migration through northern Eurasia. We confirmed the cocirculation of 2 genetically distinct viruses in a single flock of crows. The presence of H5N5 HPAIV infections in waterfowl in Japan is relatively unknown, and the lack of reports from neighboring countries on the presence of H5N5 HPAIVs from Europe has hampered the reconstruction of this genotype’s spread to eastern Asia. Continuous monitoring and rapid information sharing between countries are needed to determine the global dynamics of HPAIVs and prevent their spread.

Appendix Additional information for cocirculation of genetically distinct highly pathogenic avian influenza H5N5 and H5N1 viruses in crows, Hokkaido, Japan.
