# Supplementary material for: Cocirculation of Genetically Distinct Highly Pathogenic Avian Influenza H5N5 and H5N1 Viruses in Crows, Hokkaido, Japan
Source: Emerg Infect Dis. 2024 Sep;30(9):1912–7. doi: 10.3201/eid3009.240356 (PMC11346982; doi:10.3201/eid3009.240356)
Supplement: Appendix — Additional information for cocirculation of genetically distinct highly pathogenic avian influenza H5N5 and H5N1 viruses in crows, Hokkaido, Japan. [file 24-0356-Techapp-s1.pdf]

Article DOI: https://doi.org/10.3201/eid3009.240356

EID cannot ensure accessibility for supplementary materials supplied by authors. Readers who have difficulty accessing supplementary content should contact the authors for assistance.

Cocirculation of Genetically Distinct Highly Pathogenic Avian Influenza H5N5 and H5N1 Viruses in Crows, Hokkaido, Japan

Appendix

Additional Methods

Sample Collection and Virus Isolation

The passive surveillance of highly pathogenic avian influenza virus (HPAIV) infections in wild birds was conducted in the public garden of Sapporo City, Hokkaido. After dead crows were reported in the garden by garden staff, tracheal and cloacal swab samples were collected from the dead carcasses and immediately mixed with a virus transport medium consisting of Minimum Essential Medium (Shimadzu Corp., https://www.shimadzu.com) containing 10 mg/mL streptomycin (Meiji Seika Pharma, https://www.meiji-seika-pharma.co.jp), 10,000 U/mL penicillin G (Meiji Seika Pharma), 250 U/mL nystatin (Sigma-Aldrich, https://www.sigmaaldrich.com), 0.3 mg/mL gentamicin (MSD Co., https://www.msd.co.jp), and 0.5% bovine serum albumin fraction V (Roche, https://www.roche.com) and then inoculated into 10-day-old embryonated eggs for virus isolation (*1*). The isolation of virus was confirmed in the collected allantoic fluid by using a hemagglutination assay. The isolated viruses were subtyped by using a hemagglutination inhibition test consisting of chicken hyperimmune serum against the referenced influenza virus subtyping strain (*2*). The pathogenicity of the isolates was determined by using the virus RNA extracted from the allantoic fluid by using either TRIzol LS Reagent (Thermo Fisher Scientific, https://www.thermofisher.com) or the QIAamp Viral RNA Mini Kit (QIAGEN, https://www.qiagen.com). Direct sequencing after PCR was conducted by using a region-specific primer set to confirm the presence of nucleotides in the hemagglutinin gene encoding multiple basic amino acid residues, which is a molecular marker for HPAIV (*3*). Next-

generation sequencing was performed by using the Flongle adaptor (Oxford Nanopore Technologies, https://www.nanoporetech.com) to determine the genetic background of the HPAIV isolates; the primers used to amplify all 8 gene segments of HPAIV isolates have been previously described (*4*). Oxford nanopore libraries were prepared by using the NEB Ultra II End Repair/dA-Tailing Module (New England Biolabs, https://www.neb.com) and sequenced on the Flongle adaptor by using the Ligation Sequencing Kit V14 (Oxford Nanopore Technologies). The sequencing reads were mapped and assembled by using FluGAS version 2 (World Fusion, https://www.w-fusionus.com).

Genetic Analysis

H5 HPAIVs isolated in Hokkaido and an H5N5 isolate from Kumamoto in winter 2023–24 were used in the genetic analysis (Table 1, main text). The nucleotide sequences of H5 HPAIVs were phylogenetically analyzed by using the maximum-likelihood method and the best-fit general time-reversible model of nucleotide substitution with gamma-distribution rate variation among sites (with 4 rate categories, Γ) according to the Tamura-Nei model (*5*). Bootstrap analysis with 1,000 replications was applied to construct the phylogenetic tree in MEGA 7 (https://www.megasoftware.net) by using default parameters. Sequence data of the genes were compared with reference nucleotide sequences of representative H5Nx viruses (with different neuraminidase subtypes) belonging to clade 2.3.4.4 strains downloaded from GISAID (https://www.gisaid.org) and GenBank. BLAST (https://blast.ncbi.nlm.nih.gov) of the GISAID database was used to identify the most homologous HPAIV nucleotide sequences isolated in this study.

Antigenic Analysis

Using a cross-hemagglutination inhibition test, the antigenicity of the representative H5N5 HPAIV from Hokkaido, A/large-billed crow/Hokkaido/B073/2024 (H5N5), was compared with that of the past few seasons (2014–23) in Japan (Table 3, main text). Other antigens were analyzed and compared in representative clade 2.3.4.4 strains, including A/large-billed crow/Hokkaido/B003/2022 (Cr/Hok/B003/22; H5N2), A/chicken/Hokkaido/HU-E001/2022 (Ck/Hok/E001/22; H5N1), A/Eurasian wigeon/Q71/2022 (Ew/Hok/Q71/22; H5N1) (*6*), A/white-tailed eagle/Hokkaido/22-RU-WTE-2/2022 (WTE/Hok/R22/22; H5N1), A/duck/Vietnam/HU-16DD3/2023 (Dk/VN/HU16-DD3/23; H5N1), A/Muscovy duck/DR Congo/KAF1/2017 (Mdk/DRC/KAF1/17; H5N8) (*7*), A/chicken/Hokkaido/HU-B102/2023

(H5N1), A/Ezo red fox/Hokkaido/1/2022 (H5N1), A/large-billed crow/Hokkaido/B067/2023 (H5N1), A/northern pintail/Hokkaido/M13/2020 (H5N8), A/chicken/Kumamoto/1–7/2014 (Ck/Kum/1–7/14; H5N8) (*8*), and A/black swan/Akita/1/2016 (Bs/Aki/1/16; H5N6) (*9*), by using antiserum against Cr/Hok/B003/22 (H5N2), Ck/Hok/E001/22 (H5N1), Ew/Hok/Q71/22 (H5N1), WTE/Hok/R22/22 (H5N1), Dk/VN/HU16-DD3/23 (H5N1), Mdk/DRC/KAF1/17 (H5N8), Ck/Kum/1–7/14 (H5N8), and Bs/Aki/1/16 (H5N6) to determine cross-reactivity of hyperimmune antiserum and their corresponding antigens.

References

1. Yamamoto N, Sakoda Y, Motoshima M, Yoshino F, Soda K, Okamatsu M, et al. Characterization of a non-pathogenic H5N1 influenza virus isolated from a migratory duck flying from Siberia in Hokkaido, Japan, in October 2009. Virol J. 2011;8:65. PubMed https://doi.org/10.1186/1743-422X-8-65

2. Kida H, Yanagawa R. Isolation and characterization of influenza a viruses from wild free-flying ducks in Hokkaido, Japan. Zentralbl Bakteriol Orig A. 1979;244:135–43. PubMed

3. Kakogawa M, Onuma M, Saito K, Watanabe Y, Goka K, Asakawa M. Epidemiologic survey of avian influenza virus infection in shorebirds captured in Hokkaido, Japan. J Wildl Dis. 2020;56:651–7. PubMed https://doi.org/10.7589/2019-02-052

4. Ip HS, Uhm S, Killian ML, Torchetti MK. An evaluation of avian influenza virus whole-genome sequencing approaches using nanopore technology. Microorganisms. 2023;11:529. PubMed https://doi.org/10.3390/microorganisms11020529

5. Tamura K, Nei M. Estimation of the number of nucleotide substitutions in the control region of mitochondrial DNA in humans and chimpanzees. Mol Biol Evol. 1993;10:512–26. PubMed https://doi.org/10.1093/oxfordjournals.molbev.a040023

6. Hew LY, Isoda N, Takaya F, Ogasawara K, Kobayashi D, Huynh LT, et al. Continuous introduction of H5 high pathogenicity avian influenza viruses in Hokkaido, Japan: characterization of viruses isolated in winter 2022–2023 and early winter 2023–2024. Transbound Emerg Dis. 2024;2024:1–18. https://doi.org/10.1155/2024/1199876

7. Twabela AT, Okamatsu M, Tshilenge GM, Mpiana S, Masumu J, Nguyen LT, et al. Molecular, antigenic, and pathogenic characterization of H5N8 highly pathogenic avian influenza viruses

isolated in the Democratic Republic of Congo in 2017. Arch Virol. 2020;165:87–96. PubMed https://doi.org/10.1007/s00705-019-04456-x

8. Kanehira K, Uchida Y, Takemae N, Hikono H, Tsunekuni R, Saito T. Characterization of an H5N8 influenza A virus isolated from chickens during an outbreak of severe avian influenza in Japan in April 2014. Arch Virol. 2015;160:1629–43. PubMed https://doi.org/10.1007/s00705-015-2428-9

9. Hiono T, Okamatsu M, Matsuno K, Haga A, Iwata R, Nguyen LT, et al. Characterization of H5N6 highly pathogenic avian influenza viruses isolated from wild and captive birds in the winter season of 2016–2017 in northern Japan. Microbiol Immunol. 2017;61:387–97. PubMed https://doi.org/10.1111/1348-0421.12506

Appendix Table 1. Results of whole-genome sequencing of the highly pathogenic avian influenza virus A/large-billed crow/Hokkaido/B080/2024 (H5N1) isolated from a crow in Hokkaido, Japan, in winter 2024*

	A/large-billed crow/Hokkaido/B080/2024			
	H5N1		H5N5	
Gene segments	Coverage, %	Mean coverage	Coverage, %	Mean coverage
PB2	100	2761.018	NR	NR
PB1	100	5578.526	NR	NR
PA	100	7086.121	NR	NR
HA	100	11962.420	NR	NR
NP	100	4856.926	NR	NR
NA	100	5497.429	100	1943.684
MP	97.8	16003.720	NR	NR
NS	99.2	30145.500	NR	NR

*Sequencing was performed by using the Flongle adaptor (Oxford Nanopore Technologies, https://www.nanoporetech.com). Sequencing detected N1 and N5 NA genes in one host, indicating co-infection. HA, hemagglutinin; MP, matrix protein; NA, neuraminidase; NP, nucleoprotein; NR, no reads; NS, nonstructural protein; PA, polymerase acidic; PB1, polymerase basic 1; PB2, polymerase basic 2.

Appendix Table 2. Genetic homology between highly pathogenic avian influenza viruses A/large-billed crow/Hokkaido/B073/2024 (H5N5) (G2a subgroup) and A/large-billed crow/Hokkaido/B067/2023 (H5N1) (G2d subgroup)*

Gene	Homology, %
PB2	91.9
PB1	95.8
PA	94.8
HA	98.2
NP	96.8
NA	55.7
MP	98.7
NS	95.6

*HA, hemagglutinin; MP, matrix protein; NA, neuraminidase; NP, nucleoprotein; NS, nonstructural protein; PA, polymerase acidic; PB1, polymerase basic 1; PB2, polymerase basic 2.

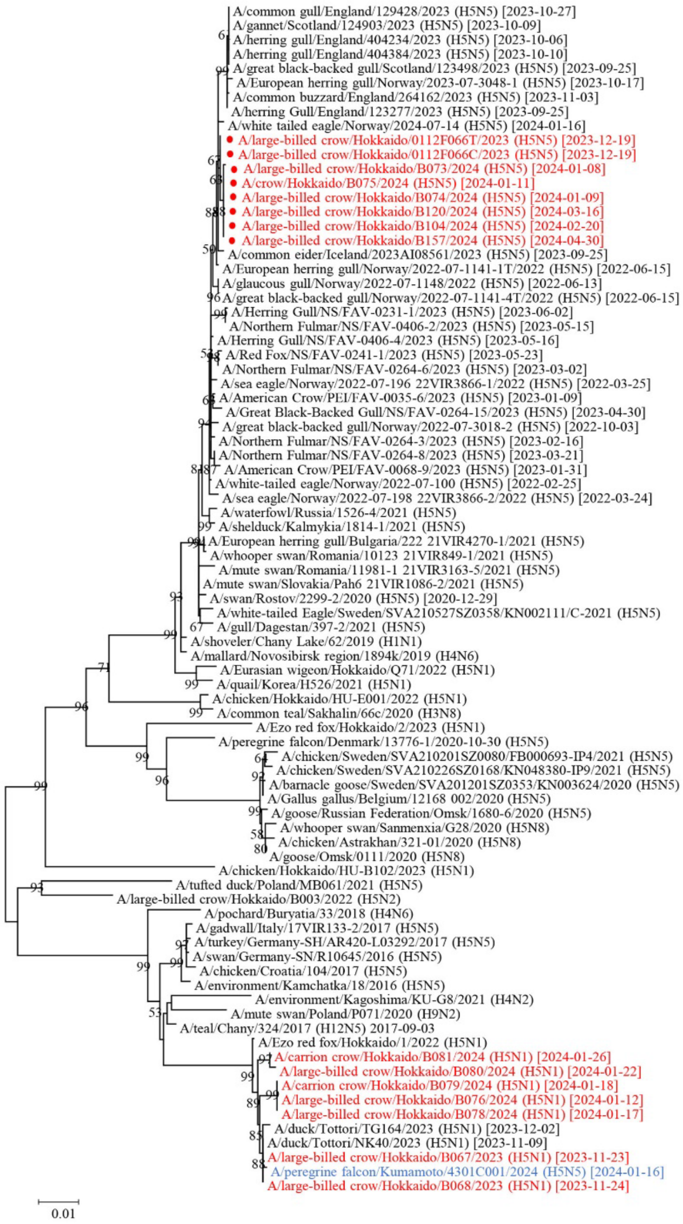

Appendix Figure 1. Phylogenetic analysis of polymerase basic 2 gene segments of H5 highly pathogenic avian influenza H5N5 and H5N1 viruses isolated in Japan in winter 2023–24. Gene segments were compared with reference strains obtained from the GISAID database (https://www.gisaid.org). Tree was constructed by using the maximum-likelihood method and MEGA 7 software (https://www.megasoftware.net). Bootstrap values (>50%) from 1,000 replicates are shown on nodes. Red text indicates H5 HPAIVs isolated from crows in Hokkaido in winter 2023–24. Blue text indicates 1 H5N5 HPAIV isolated from a peregrine falcon in Kumamato in the southern part of Japan in winter 2023–24. Red circles indicate H5N5 HPAIVs isolated in Hokkaido in winter 2023–24. Dates after strain names indicate sample collection dates for HPAIV-infected animals. Scale bar indicates number of nucleotide substitutions per site.

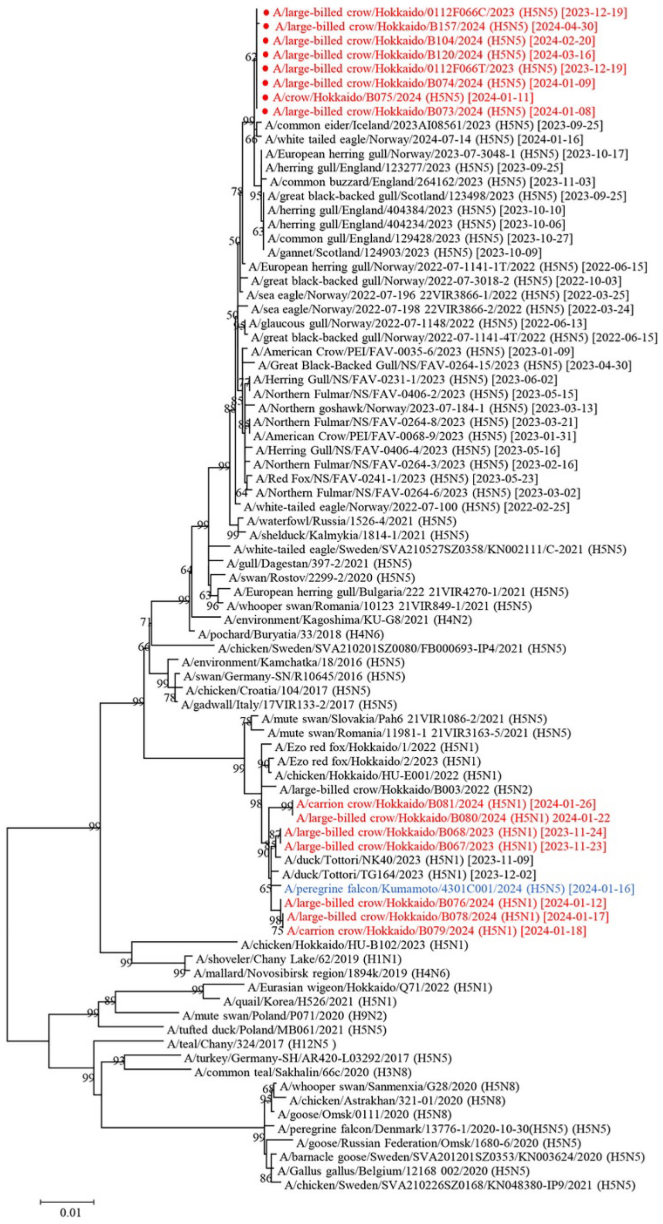

Appendix Figure 2. Phylogenetic analysis of polymerase basic 1 gene segments of H5 highly pathogenic avian influenza H5N5 and H5N1 viruses isolated in Japan in winter 2023–24. Gene segments were compared with reference strains obtained from the GISAID database (https://www.gisaid.org). Tree was constructed by using the maximum-likelihood method and MEGA 7 software (https://www.megasoftware.net). Bootstrap values (>50%) from 1,000 replicates are shown on nodes. Red text indicates H5 HPAIVs isolated from crows in Hokkaido in winter 2023–24. Blue text indicates 1 H5N5 HPAIV isolated from a peregrine falcon in Kumamato in the southern part of Japan in winter 2023–24. Red circles indicate H5N5 HPAIVs isolated in Hokkaido in winter 2023–24. Dates after strain names indicate sample collection dates for HPAIV-infected animals. Scale bar indicates number of nucleotide substitutions per site.

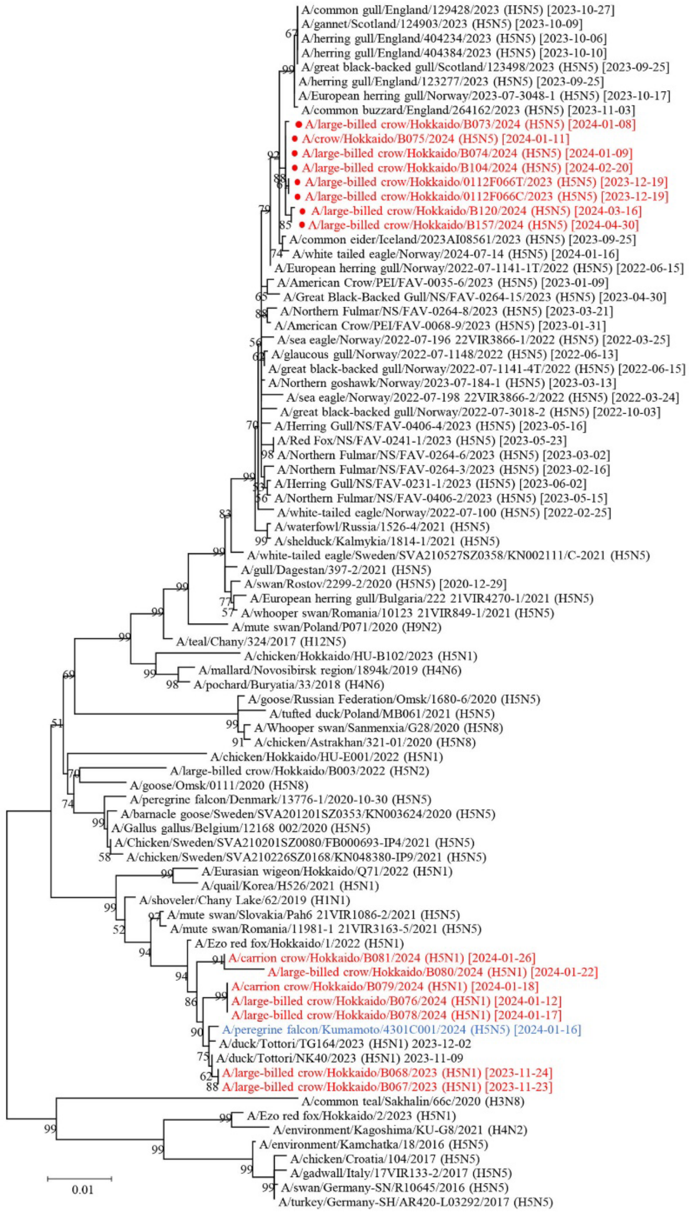

Appendix Figure 3. Phylogenetic analysis of polymerase acidic gene segments of H5 highly pathogenic avian influenza H5N5 and H5N1 viruses isolated in Japan in winter 2023–24. Gene segments were compared with reference strains obtained from the GISAID database (https://www.gisaid.org). Tree was constructed by using the maximum-likelihood method and MEGA 7 software (https://www.megasoftware.net). Bootstrap values (>50%) from 1,000 replicates are shown on nodes. Red text indicates H5 HPAIVs isolated from crows in Hokkaido in winter 2023–24. Blue text indicates 1 H5N5 HPAIV isolated from a peregrine falcon in Kumamato in the southern part of Japan in winter 2023–24. Red circles indicate H5N5 HPAIVs isolated in Hokkaido in winter 2023–24. Dates after strain names indicate sample collection dates for HPAIV-infected animals. Scale bar indicates number of nucleotide substitutions per site.

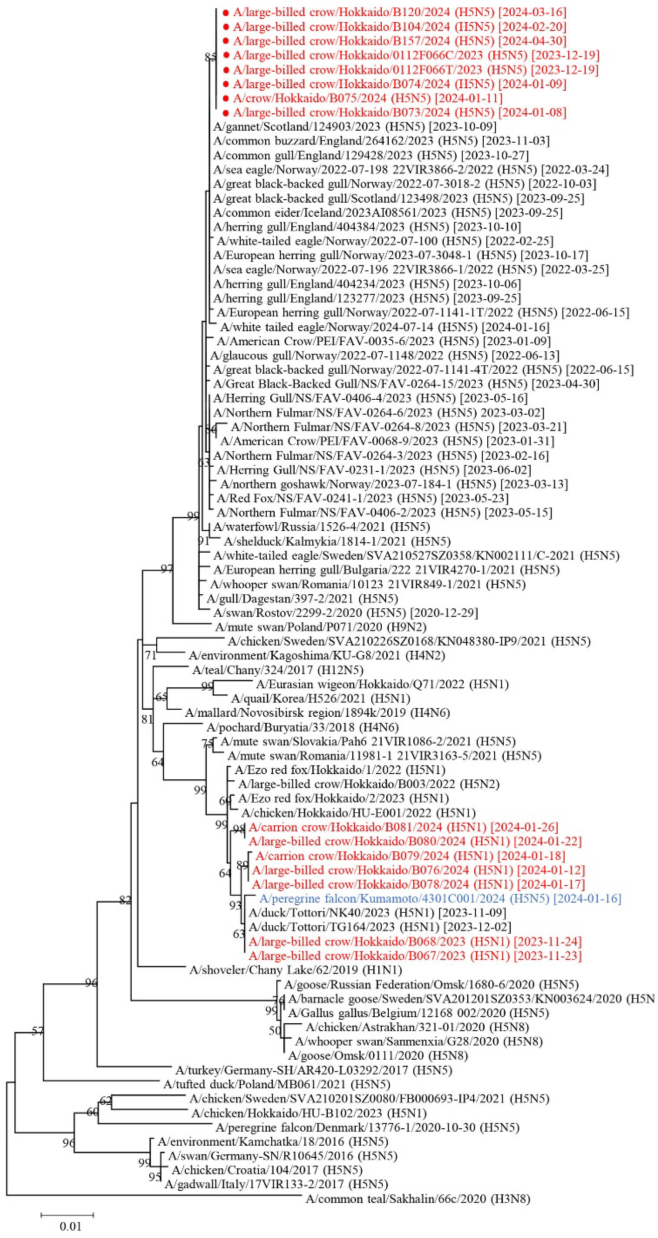

Appendix Figure 4. Phylogenetic analysis of nucleoprotein gene segments of H5 highly pathogenic avian influenza H5N5 and H5N1 viruses isolated in Japan in winter 2023–24. Gene segments were compared with reference strains obtained from the GISAID database (https://www.gisaid.org). Tree was constructed by using the maximum-likelihood method and MEGA 7 software (https://www.megasoftware.net). Bootstrap values (>50%) from 1,000 replicates are shown on nodes. Red text indicates H5 HPAIVs isolated from crows in Hokkaido in winter 2023–24. Blue text indicates 1 H5N5 HPAIV isolated from a peregrine falcon in Kumamato in the southern part of Japan in winter 2023–24. Red circles indicate H5N5 HPAIVs isolated in Hokkaido in winter 2023–24. Dates after strain names indicate sample collection dates for HPAIV-infected animals. Scale bar indicates number of nucleotide substitutions per site.

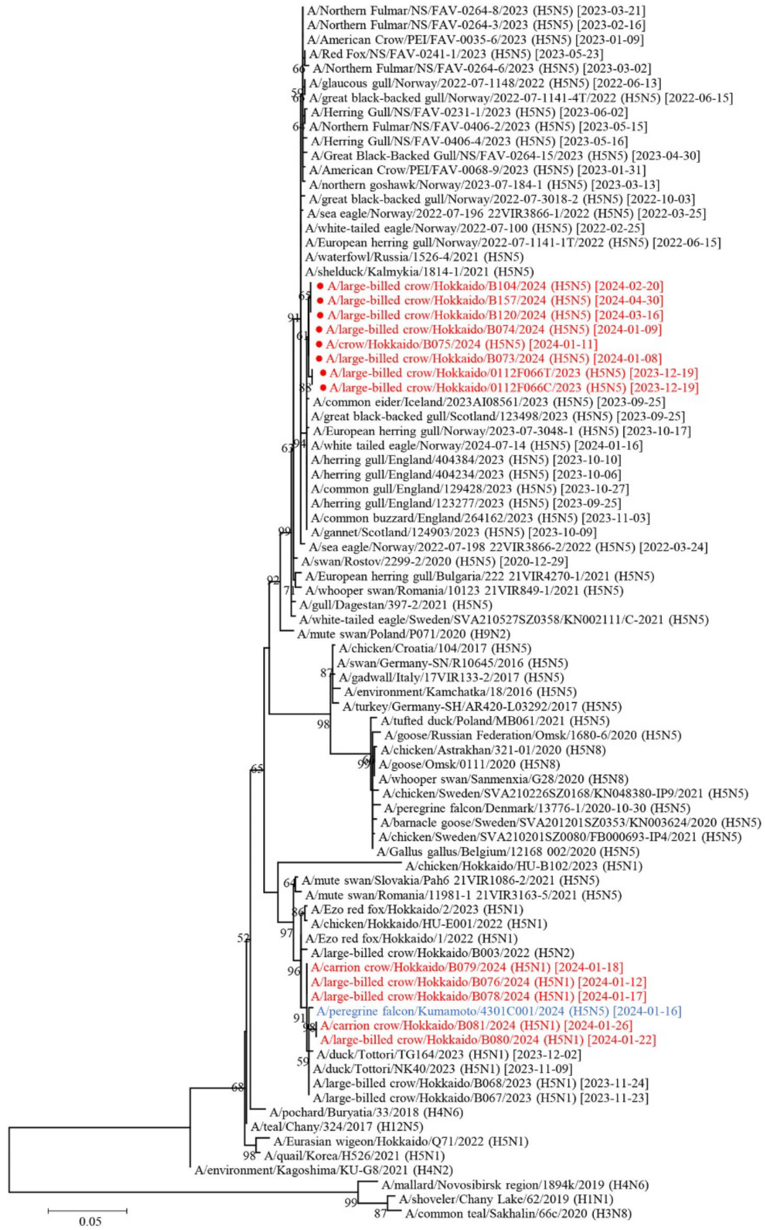

Appendix Figure 5. Phylogenetic analysis of matrix protein gene segments of H5 highly pathogenic avian influenza H5N5 and H5N1 viruses isolated in Japan in winter 2023–24. Gene segments were compared with reference strains obtained from the GISAID database (https://www.gisaid.org). Tree was constructed by using the maximum-likelihood method and MEGA 7 software (https://www.megasoftware.net). Bootstrap values (>50%) from 1,000 replicates are shown on nodes. Red text indicates H5 HPAIVs isolated from crows in Hokkaido in winter 2023–24. Blue text indicates 1 H5N5 HPAIV isolated from a peregrine falcon in Kumamato in the southern part of Japan in winter 2023–24. Red circles indicate H5N5 HPAIVs isolated in Hokkaido in winter 2023–24. Dates after strain names indicate sample collection dates for HPAIV-infected animals. Scale bar indicates number of nucleotide substitutions per site.

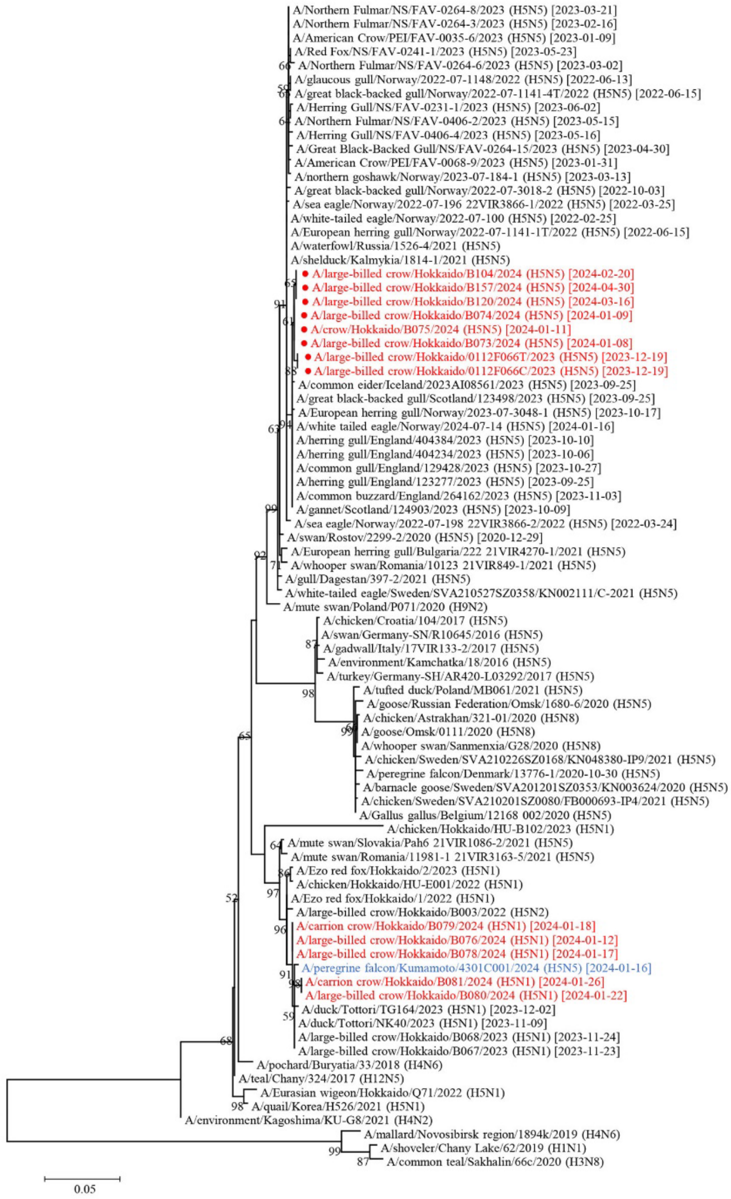

Appendix Figure 6. Phylogenetic analysis of nonstructural protein gene segments of H5 highly pathogenic avian influenza H5N5 and H5N1 viruses isolated in Japan in winter 2023–24. Gene segments were compared with reference strains obtained from the GISAID database (https://www.gisaid.org). Tree was constructed by using the maximum-likelihood method and MEGA 7 software (https://www.megasoftware.net). Bootstrap values (>50%) from 1,000 replicates are shown on nodes. Red text indicates H5 HPAIVs isolated from crows in Hokkaido in winter 2023–24. Blue text indicates 1 H5N5 HPAIV isolated from a peregrine falcon in Kumamato in the southern part of Japan in winter 2023–24. Red circles indicate H5N5 HPAIVs isolated in Hokkaido in winter 2023–24. Dates after strain names indicate sample collection dates for HPAIV-infected animals. Scale bar indicates number of nucleotide substitutions per site.
